# Optical Properties of Amorphous Carbon Thin Films Fabricated Using a High-Energy-Impulse Magnetron-Sputtering Technique

**DOI:** 10.3390/ma16217049

**Published:** 2023-11-06

**Authors:** Lukasz Skowronski, Rafal Chodun, Marek Trzcinski, Krzysztof Zdunek

**Affiliations:** 1 Division of Surface Science, Faculty of Chemical Technology and Engineering, Bydgoszcz University of Science and Technology, Kaliskiego 7, 85-796 Bydgoszcz, Poland; marek.trzcinski@pbs.edu.pl; 2 Faculty of Materials Science and Engineering, Warsaw University of Technology, Woloska 141, 02-507 Warsaw, Poland; rafal.chodun@pw.edu.pl

**Keywords:** amorphous carbon, composition, optical properties, band-gap energy

## Abstract

This paper reports the results of amorphous carbon thin films fabricated by using the gas-impulse-injection magnetron-sputtering method and differing the accelerating voltage (1.0–1.4 kV). The obtained layers were investigated using Raman spectroscopy, X-ray photoelectron spectroscopy (XRD), and spectroscopic ellipsometry (SE). The analysis of the Raman and XPS spectra point to the significant content of sp^3^ hybridization in the synthesized materials (above 54–73%). The refractive index of the films is very high—above 2.45 in the infrared spectral range. The band-gap energy (determined using the inversed-logarithmic-derivative method) depends on the discharging voltage and is in the range from 1.58 eV (785 nm) to 2.45 eV (506 nm). Based on the obtained results, we have elaborated a model explaining the a-C layers’ formation process.

## 1. Introduction

Carbon-based materials, because of their many forms, exhibit various physical and chemical properties [1,2]. They can exist as a bulk (e.g., diamond, graphite), as nanostructural systems (e.g., nanotubes, fullerenes), or layers within monolayers, i.e., graphene. Electrons from the 2s and 2p shells in the C atoms hybridize and can form sp^1^, sp^2^, and sp^3^ orbitals. Materials consisting of sp^2^ and sp^3^ carbon bonds are graphite and diamond, respectively. Amorphous carbon (a-C) is an allotropic form of C where a long-range crystalline order does not exist, in contrast to a short-range order of carbon atoms [1]. The amorphous carbon is metastable and consists of various amounts of both sp^2^ and sp^3^ bonds. Diamond-like carbon (DLC) is a special type of carbon with a significant amount of sp^3^-hybridized C atoms. Moreover, if the amount of sp^3^ bonds is greater than 80%, the tetrahedral carbon (ta-C) can be distinguished [3,4,5,6,7,8]. Properties of a-C strongly depend on the ratio of sp^2^/sp^3^; therefore, the method of fabrication and growing conditions play crucial roles during the synthesis of a-C materials [1,3,4,9,10,11,12,13,14,15,16,17,18,19,20].

Carbon coatings, due to their utilitarian properties, attract the interest of many research groups. The layers are produced by various methods, mainly physical or chemical vapor deposition methods. In the last few decades, many publications on their production and describing their properties have been published, and the interest in new/modified methods of their synthesis still continues [3,10,11,12,13,16,17,18,19]. The common factor linking these works is the connection of the composition of the layers (usually the sp^2^/sp^3^ ratio—the dominant factor) with the fabrication conditions of these materials. The analysis of the literature shows that there are three main factors determining the optoelectronic properties of carbon layers [3,10], i.e., the ratio of the number of sp^2^/sp^3^ bonds [12,13,16,17,18,19], the sp^2^ cluster size, and the distribution of sp^2^ clusters in the sp^3^ matrix [10,11].

The amorphous carbon films with a high content of sp^3^ bonds exhibit a high mechanical hardness, wear and corrosion resistance, low friction, good optical transparency, a wide band-gap, and chemical inertness [3,21,22,23]. Because of their excellent properties, these coatings are considered in a range of applications: anticorrosive [3], biomedical [24,25], optical [18,26,27], antiwear [28,29], etc. Therefore, the efficient methods enabling the synthesis of carbon films are still developed, and such modifications of them are sought which can lead to the fabrication of coatings with a high sp^3^ content. It should be emphasized that the sp^3^ content is at a very different level for the amorphous carbon coatings deposited using the magnetron-sputtering technique [12,16,18,19,30] (the sp3 content for the coatings reported in the mentioned papers is in the range from 8% to 74%).

In our research, we decided to use the high-power magnetron sputtering of a graphite target to produce DLC layers on nonpolarized and intentionally unheated substrates. Such conditions for synthesizing carbon layers correspond to the known HIPMS [31,32] mode of the magnetron operation, which seems to provide the most favorable conditions for synthesizing carbon layers with a high content of sp^3^ bonds in the carbon layer [33,34,35,36,37]. This is because the plasma produced in HIPMS is characterized by a relatively high supply of carbon vapors, a high degree of ionization resulting from the simultaneous interaction of many plasma ionization mechanisms, and an ion energy that may exceed the energy of ions produced in the dcMS mode by orders of magnitude [3,38]. Additionally, we used the GIMS magnetron-operating mode we developed, in which working gas-pressure pulses control the plasma process, oscillating periodically in the range of critical pressures [39]. This method of controlling the plasma process reduces plasma energy losses by limiting the energy dissipation of plasma particles during collisions with cold gas filling the vacuum chamber (as happens in the case of continuous gas dosing into the chamber) and their thermalization [40]. The generation of plasma for the critical pressure value for the ON valve position and its disappearance for the OFF valve position, as in the GIMS mode, can be used as a convenient way to switch ON/OFF the target current in high-power pulsed sputtering instead of using any high-power transistor as a current switcher. We also decided to place the anode at a much greater distance from the target (several cm) as a separate electrode, i.e., a departure from the standard solution, in which the anode, integrally connected to the magnetron structure, is away from the target surface (exactly from the edge of the target); a few mm at most. This solution seems to be beneficial from the point of view of increasing the energy efficiency of the plasma as a material-synthesis medium [41,42]; it can also reduce the likelihood of developing a glow discharge into an arc discharge, which may occur if the source of electric current is a capacitor charged to a voltage >1 kV.

## 2. Materials and Methods

### 2.1. Sample Preparation

**Figure 1:**
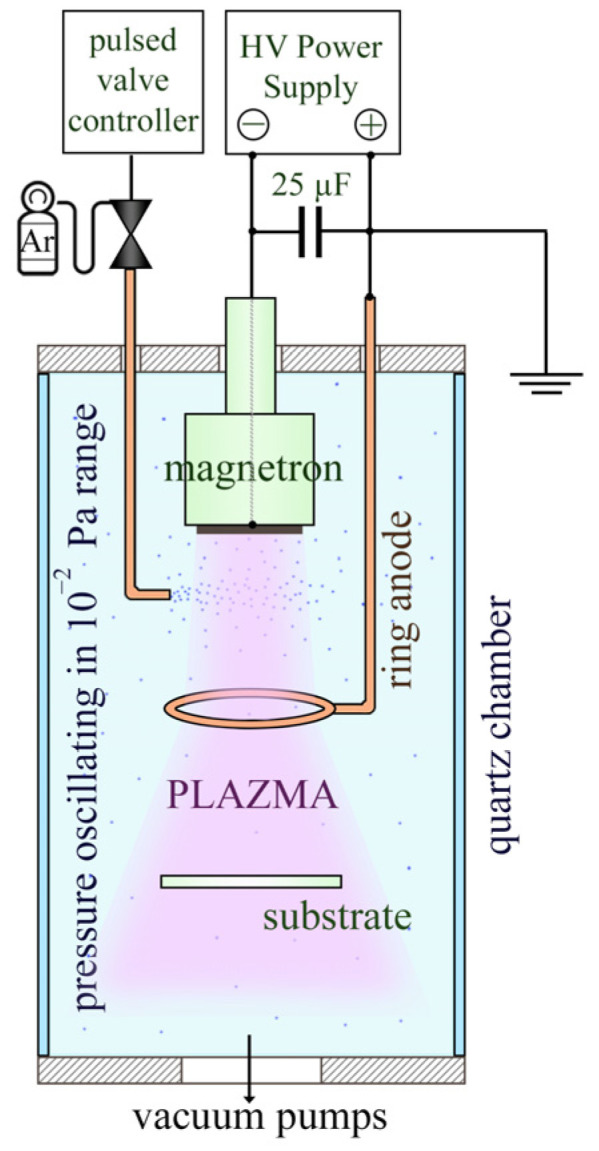
Diagram of the apparatus used in the experiment.

The a-C thin films were fabricated using the magnetron-sputtering method. The graphite target (a diameter 50 mm; a thickness 4 mm; a purity 99.97%) was used during the deposition process. The sputtering process was carried out in Ar atmosphere (5N). The sputtering gas was periodically (0.5 Hz) injected to the vacuum chamber directly near to the target. The plasma-generation process was controlled by a millisecond pulse valve (the ON/OFF regime) and a rapid increase/decrease of the pressure of the Ar (from/to ~0.2 × 10^−2^ Pa to/from ~5.0 × 10^−2^ Pa), wherein the opening time of the valve was 3 ms (ON). After closing the valve (OFF), the pressure in the chamber dropped to its base value, and the gas discharging was stopped. The time of the deposition was 1000 s. The nonbalanced magnetron target (the graphite cathode) was connected to the GND of the device. The anode was made from copper pipe of a thickness of 10 mm and a diameter 90 mm, and was connected to the 25 µF condenser charged from the DC power supply (DPS, Dora Power Systems, Wroclaw, Poland) to voltages of 1.0 kV, 1.2 kV, and 1.4 kV. The a-C films were deposited on silicon (100). The target-substrate distance was 100 mm. The samples produced were marked according the voltage used during the deposition (aC1.0, aC1.2, and aC1.4). The experimental set-up is presented in Figure 1.

### 2.2. Sample Characterization

The vibrational spectroscopy of the fabricated amorphous carbon films was performed using a 2.33 eV Ar^+^ VIS laser (532 nm) and a 4.66 eV UV laser (266 nm). The FQCW266-50 diode-pumped continuous-wave solid-state laser (Crylas, Fremont, CA, USA) was used as a source for the UV excitation. The JASCO NRS 5100 Raman spectrometer (JASCO, Tokyo, Japan) working in backscattering mode was used to disperse scattered light. The lasers’ ~5 mW laser beams were focused onto ~20 μm spots on the surface of the films using the 100× (VIS) and 40× (UV) objectives. The parameters of spectra registration were optimized based on the peak/noise ratio of the registered spectra. The registered spectra were processed using Spectra Manager Micro Imaging Analysis v.2.3 (JASCO, Tokyo, Japan) and Curve Fitting v 1.9 (JASCO, Tokyo, Japan) spectra-processing software. Vibrational spectra were interpolated, and their background was subtracted to realign intensity levels at each side of the presented range. The curve-fitting procedure determined the G and D peak positions and full width at half-maximum FWHM. The curve-fitting optimized the intensities, positions, FWHM, and shapes (Gaussian, Lorentzian, or mixed Gaussian–Lorentzian function) of any vibrational components of the registered spectra using a least-squares method. The sp^3^ bond content was evaluated based on G peak parameters: the G peak FWHM and G peak dispersion rate [43]. The sp^3^/sp^2^ ratio as the G peak dispersion is expressed by:

(1) $${sp}^{3}content=-0.07+2.5\times Disp(G),$$

where:

(2) $$Disp\left(G\right)=\left|\left.\frac{Pos\left(G\right)@\lambda_{2}-Pos\left(G\right)@\lambda_{1}}{\lambda_{2}-\lambda_{1}}\right|\right.,$$

$Pos\left(G\right)@\lambda_{2}$ and $Pos\left(G\right)@\lambda_{1}$ are the *G* peak positions at $\lambda_{2}$ and $\lambda_{1}$ irradiations, respectively, $\lambda_{2}$ and $\lambda_{1}$ are laser wavelengths.

The sp^3^/sp^2^ ratio as the FWHM criterion can be calculated from:

(3) $${sp}^{3}content=-0.25+1.9\times 10^{-2}\times W-3.01\times 10^{-5}\times W^{2},$$

where:

(4) $$W=FWHM\left(G\right)+0.21\times (514-\lambda ),$$

and λ is the laser wavelength.

Spectroscopic ellipsometry (SE) measurements were performed using the V-VASE instrument from J.A.Woollam Co., Inc. (Lincoln, NE, USA). The Ψ and Δ ellipsometric azimuths are defined as [44,45]:

(5) $$\tilde{\rho }=\tan\Psi \exp i\Delta ,$$

where $\tilde{\rho }$ is the ratio of the amplitude reflection of the electromagnetic radiation for p- and s- polarization, and i is the imaginary unit, and were measured in the UV–vis–NIR spectral range (0.62–6.5 eV, 2000–190 nm) for three angles of incidence: 65°, 70°, and 75°. The WASE32 software (version 3.774) was used to analyze the ellipsometric data. 

The X-ray photoelectron spectroscopy (XPS) technique was used to investigate the contribution of the sp^2^ and sp^3^ hybridization bonds in the near-surface layers of the samples. Measurements were performed under ultrahigh-vacuum conditions (base pressure ≤ 2 × 10^−10^ mbar). The excitation radiation source was an Al K_α_ lamp (1486.6 eV). The angle of incidence of the excitation beam was 55°. The energy of the photoelectrons was analyzed using the VG-Scienta R3000 (Uppsala, Sweden) analyser (ΔE = 100 meV). The registered data were fitted by means of the CasaXPS software (Version 2.3.16). The Shirley background and Gauss–Lorentz line shapes were used during the deconvolution of particular signals.

## 3. Results and Discussion

### 3.1. The Composition of the Fabricated a-C Films

**Figure 2:**
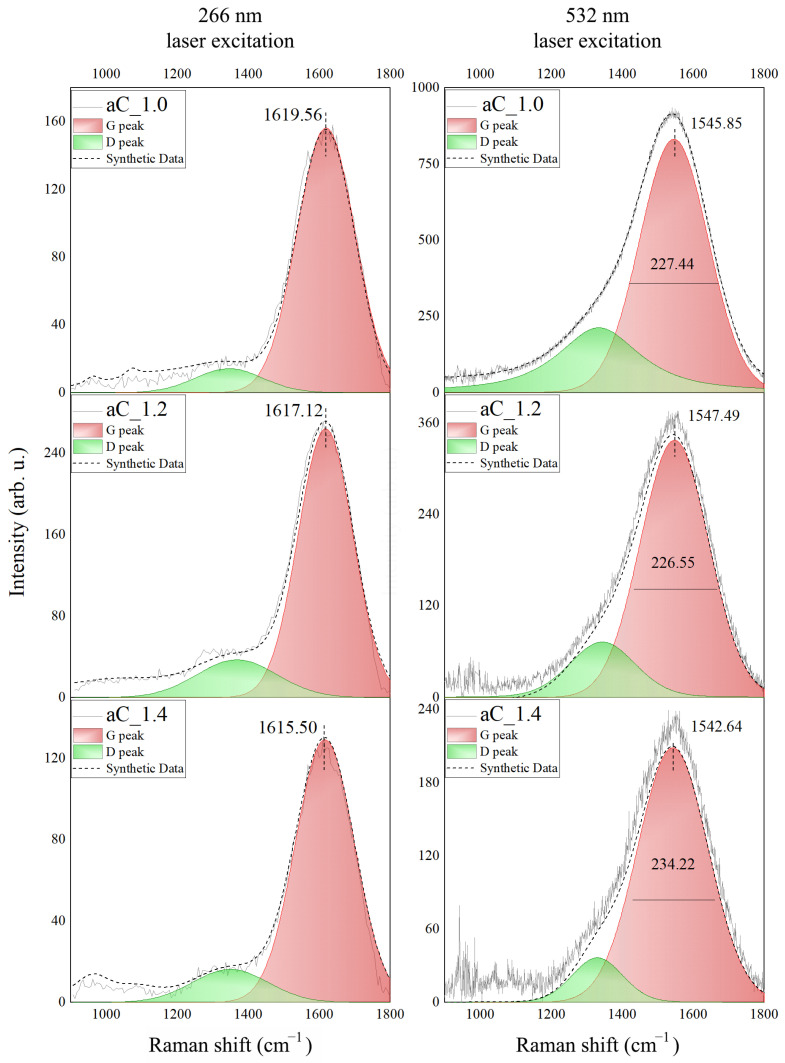
The Raman spectra registered for the a-C thin films irradiated by 266 nm (**left**) and 532 nm (**right**) lasers and fitted with D and G elementary spectra.

The Raman spectra recorded for the fabricated amorphous carbon thin films are presented in Figure 2. The details (position, FWHM, and intensity) of the Raman spectra are summarized in Table A1 (see Appendix A). The D peak is observed at 1300–1400 cm^−1^, while the G peak is at 1550–1620 cm^−1^, wherein the G peak exhibits a significantly higher intensity than that for the D one. The exact position and FWHM of the G peak were used to estimate the content of the sp^3^ bonds in the deposited films based on the procedure described in [45] (sp^3^ content G_FWHM_) and using the method based on the position of the peak (sp^3^ content G_Pos._). The obtained results are summarized in Table 1. The sp^3^ content in the fabricated a-C thin films is in the range from 58% to 73%, depending on the sample and the method of estimation. However, taking into account the uncertainty of the estimation (which is 6–8% [45]), we can state that there is no significant difference in the composition of these samples, or that the differences are within the estimated error range. It should be emphasized that, despite the quite high value of the uncertainty, the sp^3^ content is at a very high level for the amorphous carbon materials deposited using the magnetron-sputtering technique [12,16,18,19,31]. 

**Figure 3:**
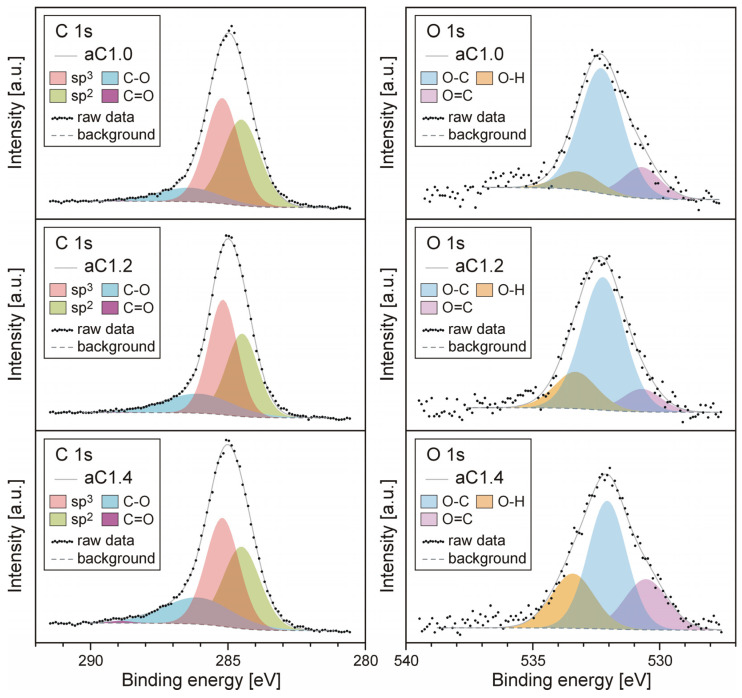
C1s and O1s XPS spectra of the fabricated a-C films.

The XPS measurements (see Figure 3) indicate that only carbon and oxygen are observed on the surface of the samples. The main components of the carbon peak come from sp^3^- and sp^2^-hybridized bonds. One may find them at energies of approximately 285.2 and 284.5 eV for sp^3^ and sp^2^ [46,47], respectively. The estimated sp^3^ bond content determined by the XPS method is slightly lower than the estimates achieved by the analysis of Raman spectra. It is pretty often reported in the literature that the surface layer of carbon coatings deposited by plasma-surface-engineering methods is enriched in sp^2^ bonds [48]; therefore, an XPS study considering only the material at its surface will always show increased sp^2^ content compared to methods sensitive to material features from a greater depth (Raman spectroscopy). In all of the samples, the C1s level is broadened towards higher binding energies. This is related to the presence of hydroxyl bonds (C-O) on the surface [49], and also, although to a much lesser extent, carboxyl bonds (C=O) [49]. These levels were determined at binding energies of 286.2 and 288.9 eV, respectively.

The oxygen peak originating from the O1s level has a much lower intensity than C1s in all samples, but is relatively broad. It can be deconvoluted using three components. The most intense of them, found at an energy of 532.2 eV, comes from O-C bonds [49]. The peak from the oxygen atoms involved in O=C carboxyl bonds is found at an energy of approximately 530.7 eV [49]. The third component, occurring at around 533.3 eV, most likely comes from O-H bonds [49]. The deconvoluted XPS spectra confirm that, in all samples, the share of carbon diamond bonds (sp^3^) is relatively high and, compared to graphite bonds (sp^2^), is estimated to be approximately 55%. The XPS analysis did not show, however, any significant differences between the tested samples. The main difference is the total oxygen content. In sample aC1.0, the surface atomic concentration of oxygen was estimated at 8.8%, while in aC1.2, it was 6.2%, and in aC1.4, only 4.7%.

### 3.2. Optical Properties of the a-C Films

**Figure 4:**
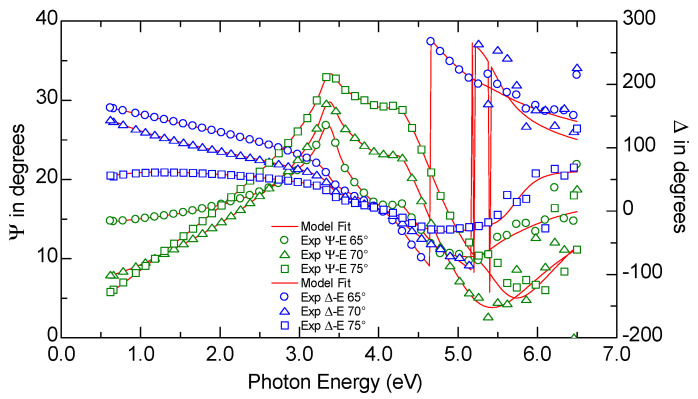
Measured and calculated Ψ and Δ ellipsometric azimuths for the aC1.0 sample (for the experimental data, every fifth data point has been plotted). The MSE value of the fit was 1.39.

The optical constants and thicknesses of the amorphous carbon thin films have been determined using a four-medium optical model of a sample (from bottom to top): 1—Si substrate, 2—native SiO_2_, 3—a-C film, and 4—ambient. The optical constants of Si and SiO_2_ were taken from the database [44]. The complex refractive index of the a-C films ($\tilde{n}$) was parameterized using a sum of the high-frequency dielectric constant ($\varepsilon_{\infty }$ = 1) and Lorentzian oscillators [44,45]:

(6) $${\tilde{n}}^{2}={\left(n+ik\right)}^{2}=\varepsilon_{\infty }+\sum_{j=1}^{m}\frac{A_{j}E_{j}^{2}}{E_{j}^{2}-E_{}^{2}-i{Br}_{j}E},$$

where *n* and *k* are the real part of the complex refractive index (or, shortly, the refractive index) and the extinction coefficient, respectively. In Equation (6), the parameters of the *j*-th Lorentzian oscillator are amplitude (*A_j_*), energy (*E_j_*), and broadening (*Br_j_*). The fitting parameters were changed to minimize the standard mean squared error (MSE). The mathematical formula of the MSE can be found in [44,45]. The example of experimental and calculated ellipsometric azimuths (for the aC1.0 sample) are presented in Figure 4. The values of the MSE for all of the fits are below five (see Table 2), which proves that the fitting procedure has been carried out correctly.

**Figure 5:**
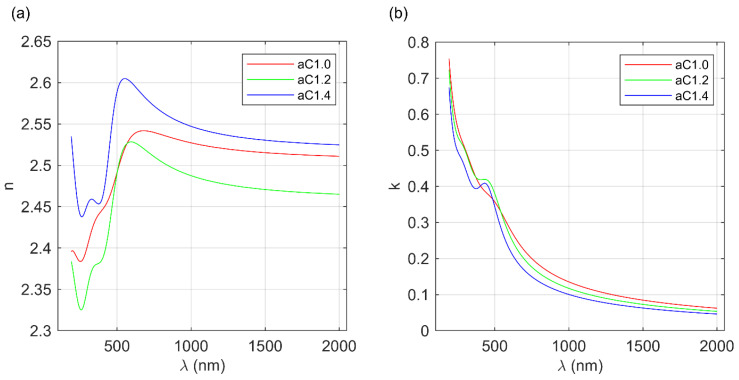
(**a**) Real part of the complex refractive index and (**b**) extinction coefficient of the carbon films.

The thicknesses of the fabricated a-C films are summarized in Table 2, and are equal to 32.1 ± 0.4 nm, 22.1 ± 0.4 nm, and 21.5 ± 0.3 nm for aC1.0, aC1.2, and aC1.4 carbon layers, respectively. The optical constants of the deposited amorphous carbon layers (the real part of the complex refractive index—*n* and the extinction coefficient—*k*) are shown in Figure 5. The shape of the refractive index for all of the carbon films (Figure 5a) is similar. In the infrared (IR) spectral range, the normal dispersion can be observed, i.e., a decrease in the refractive index value with the increasing wavelength. This behavior of n is typical for dielectric materials [50]. In the visible (vis) and in the ultraviolet (UV) spectral ranges of electromagnetic radiation, the absorption bands can be observed. Values of the refractive index in IR are in the range from 2.45 to 2.55, wherein the lowest values have been obtained for the aC1.2 sample and the highest for the aC1.0 film. Generally, the refractive index of carbon layers in the IR spectral range depends on the density of the fabricated coating (which can be in the range from 1.6 to 3.5 g/cm^3^), and is in the range from 1.5 to 2.6 [3,30]. The obtained values of n are evidence that the deposited a-C thin films are dense. 

**Figure 6:**
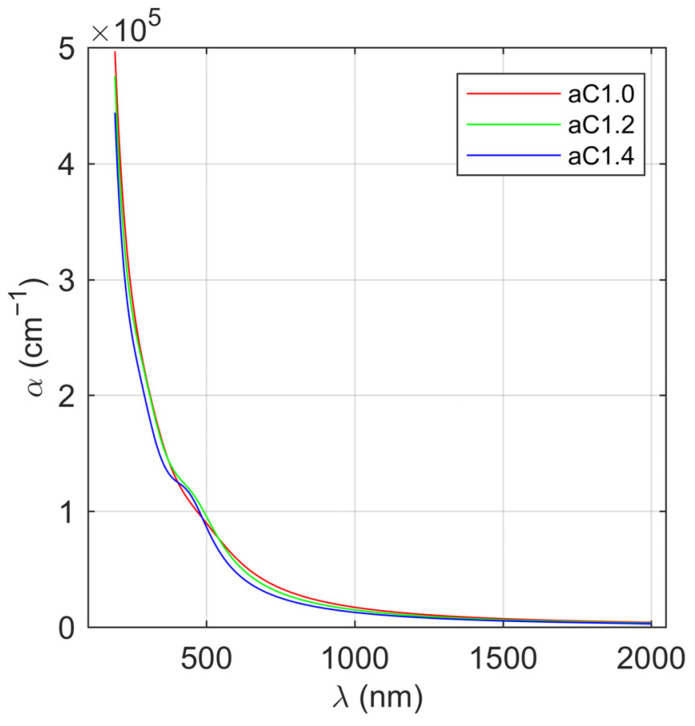
Absorption coefficient of the deposited a-C thin layers.

The extinction coefficient (see Figure 5b) increases with the decrease in the wavelength. In the IR spectral range, the values of *k* for all of the analyzed a-C thin films demonstrate low (*k* < 0.2), however, nonzero, values. Therefore, this spectral region can be described as weakly absorbing. In the vis and UV range, the extinction coefficient values increase to (depending on the sample) 0.65–0.75. Two absorption bands are visible for the fabricated amorphous carbon layers in the spectral range of 190–2000 nm: the first one at about 400–500 nm (for the sample aC1.0, it is screened by the second band appearing in UV) and the second below 190 nm. Only an edge of this band is visible in Figure 5b, because its maximum lies outside the measuring range. Based on the determined extinction coefficient (see Figure 5b), the absorption coefficient (*α*) has been calculated (see Figure 6) using the following formula [45]:

(7) $$\alpha =\frac{4\pi k}{\lambda },$$

where *λ* is the wavelength of the incident light. In the weakly absorbing spectral range (IR), the values of α do not exceed 0.5 × 10^5^ cm^−1^. For shorter wavelengths, the absorption coefficient increases rapidly by one order of magnitude, and in the UV spectral range, reaches value of about 5 × 10^5^ cm^−1^.

**Figure 7:**
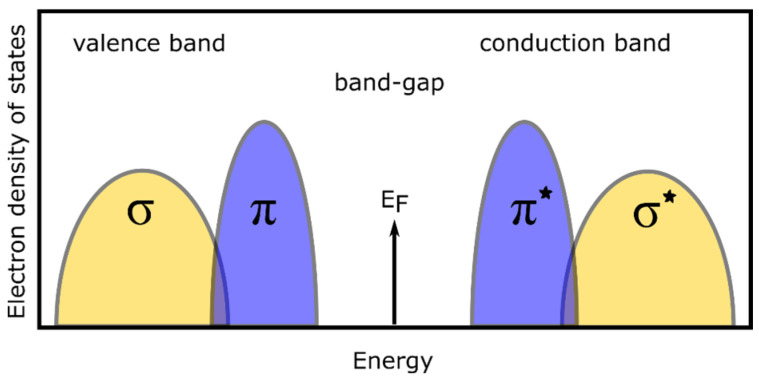
Density of electronic states for C-based materials.

Depending on the method and synthesis conditions of the carbon layers, the obtained material is generally built of carbon atoms with sp^2^ and/or sp^3^ hybridization, and its properties (including optical) strongly depend on the bonds between the C atoms. The optical properties of the carbon layers can be explained based on the schematic shown in Figure 7; the electron density of states. For the diamond (the bonds between C atoms exhibit sp^3^ hybridization), only σ bonds are formed, while in the case of materials containing C atoms with both sp^2^ and sp^3^ hybridization, additional π bonds are formed.

Diamond (containing only σ bonds) has an energy gap of 5.5 eV (225 nm), corresponding to the σ → σ* transitions [3,7], wherein it is an indirect band-gap [3]. With the existence of defects and/or doping [51,52], the energy of the band-gap may be decreased. The appearance of sp^2^ bonds in the diamond structure results in the formation of strongly localized π states, and thus the possibility of π → π* electron transitions. An increase in the concentration of sp^2^-hybridized C atoms leads to an increase in the intensity of the π → π* electron transition. As a result, the band-gap energy decreases [52,53]; this is observed as well as an increase in the extinction (and absorption) coefficient in this spectral range. In the measured spectral range of 0.6–6.5. eV (200–2000 nm), the shape of the extinction coefficient (as well as the absorption coefficient) has two components. The first of them is related to the electronic transitions π → π* (maximum at 4–4.5 eV/310–275 nm) [30,54]. The second, related to the σ → σ* transition, has a maximum outside the considered spectral range (10–16 eV) [3,30].

The standard method of determining the value of the band-gap energy is based on the Tauc plot (e.g., [55,56]). The relationship (*αhν*)^1/*m*^ is plotted as a function of the incident photon energy (*hν*). The *m* coefficient is a parameter that determines the type of electronic transition, and takes the following values [55]: *m* = 1/2 for an allowed direct transition, *m* = 3/2 for a forbidden direct transition, *m* = 2 for an allowed indirect transition, and *m* = 3 for a forbidden indirect transition. In the Tauc method, a specific type of transition should be assumed ‘a priori’, and thus the value of the m coefficient. This assumption may be incorrect for newly synthesized compounds, or materials with two phases or a multiphase structure, in which individual components are characterized by different optical properties (i.e., a different kind of electronic transition). In such measurements, the material is usually tested as a whole, and its properties should be treated as the effective ones (not as the properties of individual components). Therefore, to determine both the energy gap and the type of electronic transition, the inverse logarithmic derivative (ILD) method presented in [56] was used. This method allows for the independent determination of both the value of the energy gap *E_g_* and the value of the m parameter based on the gradient and the energy axis intercept of the equation [56]:

(8) $$\frac{∆ln\left(h\nu \right)}{∆ln\left(\alpha h\nu \right)}=\frac{1}{m}\left(h\nu -E_{g}\right),$$

**Figure 8:**
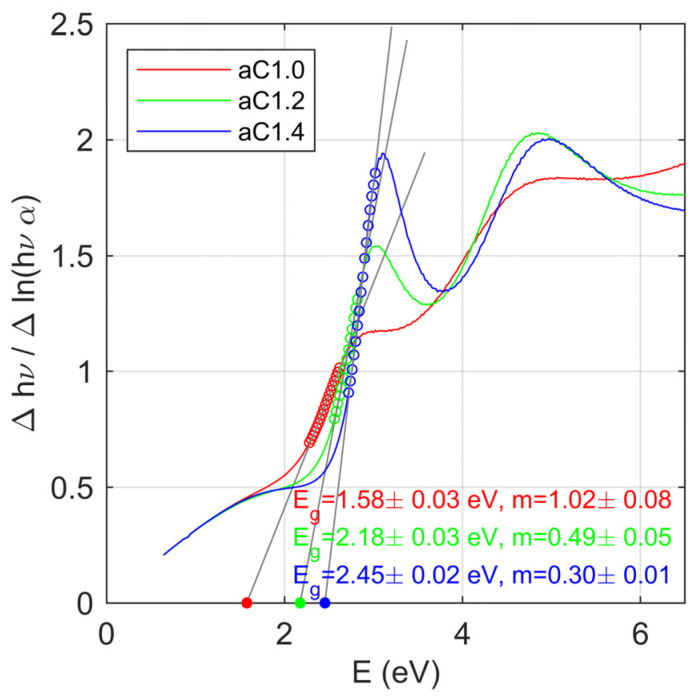
The ILD plot of the carbon films.

The ILD plot for the fabricated a-C films is presented in Figure 8. The determined values of the energy gap *E_g_* and the *m* coefficient are summarized in Table 3. For the aC1.0 sample, the band-gap energy lies in the near-infrared spectral range (*E_g_* = 1.58 ± 0.03 eV; 785 ± 20 nm) and the *m* coefficient is about 1 (*m* = 1.02 ± 0.02). For the other samples (aC1.2 and aC1.4), the band-gap energy is in the visible spectral range. For the aC1.2 thin film, *E_g_* = 2.18 ± 0.03 eV (569 ± 8 nm), and for aC1.4, *E_g_* = 2.45 ± 0.02 eV (506 ± 5 nm). The value of the *m* coefficient for these films is two to three times lower than that estimated for the aC1.0 carbon layer. The determined values of the *m* parameter indicate the direct band-gap for the aC1.2 and aC1.4 films, while the aC1.0 sample contains fractions responsible for both direct and indirect transitions.

### 3.3. Structure of the a-C Films

The analysis of Raman spectra indicates that the sp^3^-bonded carbon atom content is very high (58–73% depending on the method of calculation); however, taking into account the uncertainty of this estimation, we can conclude that there is no significant difference between these a-C films. The estimated content of sp^3^-hybridized C atoms using the XPS technique is lower (about 55%) than that estimated using the vibrational spectroscopy. The XPS technique is a surface-sensitive method and, in contrast to the Raman measurements, where the recorded signal comes from the whole film, provides us with information only about the surface (subsurface) state of the sample. Such discrepancies between the composition of the carbon films were observed earlier [48,57,58]. Optical measurements show a significant difference in the properties of the fabricated a-C films. The band-gap energy increases from 1.58 eV (785 nm) to 2.45 eV (506 nm) with the increase in the condenser charging voltage from 1.0 kV to 1.4 kV, respectively. The plasma-creation conditions (the high-energetic process with the maintaining of the kinetic energy of the plasma-phase particles achieved by the impulse injection of the Ar gas [59]) during the synthesis of the amorphous carbon films provided the nucleation onto ions, combined with the energy transfer from plasma to ultrasmall C clusters, caused by their inelastic collisions with electrons, whose energy can be similar to the energy of C bonds (including sp^3^ bonds). The intensity of these phenomena increase with the increase in the voltage discharging (the energy is proportional to the squared voltage), and thus the probability of the sp^3^-hybridized C bond creation. Simultaneously, with the increase of the discharging voltage, the increase in the kinetic energy of particles bombarding the substrate is observed. This process leads to the degradation of sp^3^ bonds to amorphized and degraded sp^3^/sp^2^ regions. The spectroscopic ellipsometry technique is sensitive to the quantity of the sp^3^ bonds (e.g., shows higher band-gap energy for the largest discharging voltage), while Raman and XPS measurements are quality-sensitive techniques which show the subtle differences in the structures of the produced a-C films. 

The pulsed plasma used in our experiments meets the requirements resulting from the concept of homogeneous nucleation on ions, which explains the mechanism of creating carbon bonds with an sp^3^ hybrid [60,61]. According to this concept, in carbon clusters formed in the plasma, which may be critical nuclei due to their charge as a result of inelastic collisions with plasma electrons (nonequilibrium), valence electrons may be excited, which favors the creation of sp^3^ bonds at the expense of sp^2^ bonds. The ultrasmall size of critical nuclei limits the dissipation of energy obtained from electrons into phonon excitations. After reaching the substrate, the clusters condense on its surface, creating a carbon layer rich in sp^3^ hybrid bonds, and the cold substrate stabilizes the state of the thermodynamic metastability of the carbon layer. In our opinion, the fact of obtaining a relatively high content of sp^3^ bonds in the layers we produce can be interpreted following the concept mentioned above, especially since it has already been convincingly verified positively in the case of the synthesis of DLC layers made from high-power pulsed plasma generated in a coaxial accelerator [29,62]. However, we do not reject the subplantation concept commonly accepted in the literature, according to which the creation of sp^3^ carbon bonds takes place in the surface zone of the in situ growing carbon layer through the subplantation of energetic carbon ions (ca. 100 eV) in this zone, and the formation of a thermobaric wave around the track. As a result of the accompanying deformations and stresses, the sp^2^ bond networks in the amorphous carbon network may transform into sp^3^ bonds [63,64]. However, it seems to us, due to the lack of the electrical polarization of the substrate mentioned in the simulation concept as a condition for providing carbon ions the necessary kinetic energy and content of sp^3^ hybrid bonds as high as 60% and more in our DLC layers, that the idea of homogeneous nucleation in plasma may be more adequate in our case, especially if it is related to the explanation of the formation of sp^3^ hybrid bonds. In turn, the adequacy of the subplantation concept can be considered in the context that the content of sp^3^ hybrid bonds decreases with the increasing discharge voltage of the capacitor. The probable increase in the energy of carbon ions accompanying this may cause the secondary graphitization of the DLC layer due to the too-intense dissipation of the kinetic energy of the ions bombarding it.

## 4. Conclusions

The amorphous carbon thin films were fabricated by using the gas-impulse-injection magnetron-sputtering method for three values of the accelerating voltage (1.0, 1.2, and 1.4 kV). The composition of the layers was investigated using Raman spectroscopy and X-ray photoelectron spectroscopy. Based on these experimental techniques, the sp^3^ hybridization content in the synthesized materials was estimated. For all of the a-C films, the content is above 60%, which is significantly higher than that reported for amorphous carbon films fabricated using magnetron-sputtering techniques. The spectroscopic ellipsometry measurements were performed to determine both the thicknesses (~20–30 nm) of the carbon films as well as their optical constants. The refractive index in the NIR spectral range exhibits the normal dispersion. Its high value (above 2.45 in NIR) indicates that the materials are dense. The band-gap energy increases from 1.58 eV (785 nm) through 2.18 eV (569 nm) to 2.45 eV (506 nm) with the increase in the accelerating voltage from 1.0 kV through 1.2 kV to 1.4 kV. In the UV–vis spectral range, the absorption bands are visible. The first one can be attributed to π → π* and the second one to the σ → σ* electron transitions. However, for the second transition in the UV spectral range, only the edge of the absorption band is visible.

## Figures and Tables

**Table 1:** The fraction of sp^3^ bonds in the a-C layers estimated based on the G peak in the Raman spectra and on the XPS deconvolution of the C1s peak.

Sample	sp^3^ Content		
	G_FWHM_	G_Pos._	XPS
aC1.0	62 ± 6%	69 ± 8%	55 ± 3%
aC1.2	58 ± 6%	68 ± 8%	56 ± 3%
aC1.4	61 ± 6%	73 ± 8%	54 ± 6%

**Table 2:** Thicknesses and the root mean squared error (MSE) determined for the analyzed samples.

Sample	d (nm)	MSE (-)
aC1.0	32.1 ± 0.4	1.39
aC1.2	22.1 ± 0.4	4.31
aC1.4	21.5 ± 0.3	2.75

**Table 3:** Band-gap energy (E_g_) and coefficient m.

Sample	*E_g_* (eV)	*E_g_* (nm)	*m* (-)
aC1.0	1.58 ± 0.03	785 ± 20 nm	1.02 ± 0.02
aC1.2	2.18 ± 0.03	569 ± 8 nm	0.49 ± 0.05
aC1.4	2.45 ± 0.02	506 ± 5 nm	0.30 ± 0.01

## Data Availability

Data are available from the corresponding author.

## Appendix A

**Table A1:** The parameters of Raman spectra: position, FWHM, and intensity of G and D peaks (the excitation wavelength: 266 nm and 532 nm), as well as the sp^3^ content calculated based on the method presented in [43] (sp^3^ content G_FWHM_) and on the intensity of the G and D peaks (sp^3^ content G_Int._).

Sample	Peak	Position (cm^−1^)	FWHM (cm^−1^)	Intensity (arb.u.)	sp^3^ Content G_FWHM_ (%)	sp^3^ Content G_Pos_. (%)
aC1.0	G_266_	1619.56	200.41	147.58	62 ± 6	69 ± 8
	D_266_	1413.24	212.00	18.05		
	G_532_	1545.85	227.44	831.57		
	D_532_	1334.02	276.16	213.26		
aC1.2	G_266_	1617.18	185.18	249.54	58 ± 6	69 ± 8
	D_266_	1413.39	192.40	43.09		
	G_532_	1547.49	226.55	337.40		
	D_532_	1343.94	219.20	72.65		
aC1.4	G_266_	1615.50	189.53	120.35	61 ± 6	73 ± 8
	D_266_	1410.11	172.52	17.65		
	G_532_	1542.64	234.22	208.67		
	D_532_	1329.93	167.84	36.66		
